# Supplementary figures and images for: Pilot-scale depuration demonstrates the suitability of non-pathogenic Vibrio parahaemolyticus as a surrogate for commercial-scale validation studies
Source: PLoS One. 2025 Oct 8;20(10):e0334240. doi: 10.1371/journal.pone.0334240 (PMC12507247; doi:10.1371/journal.pone.0334240)

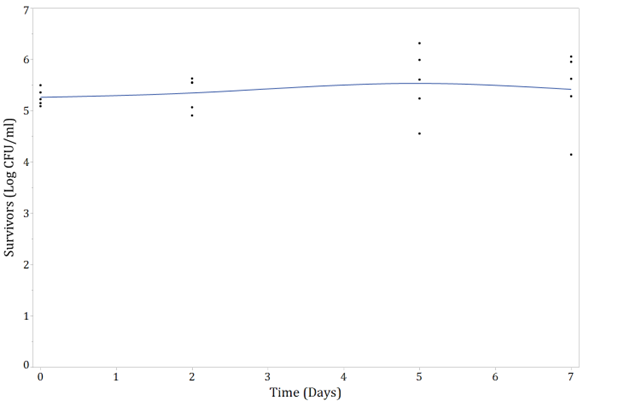

Supplement: S1 Fig — Day 0 samples were taken after oysters were held in contaminated water for 24 hrs. (TIFF) [file pone.0334240.s001.tiff]
